# Bone marrow stromal cell-derived hepcidin has antimicrobial and immunomodulatory activities

**DOI:** 10.1038/s41598-024-54227-1

**Published:** 2024-02-17

**Authors:** Miklós Krepuska, Balázs Mayer, Lynn Vitale-Cross, Vamsee D. Myneni, Michael K. Boyajian, Krisztián Németh, Ildikó Szalayova, Ted Cho, Ian McClain-Caldwell, Aaron D. Gingerich, Huiling Han, Mark Westerman, Balázs Rada, Éva Mezey

**Affiliations:** 1 National Institutes of Health, NIDCR, ASCS, Bethesda, MD USA; 2 Department of Neuroradiology, University Hospital Zürich, Zürich, Switzerland; 3 Stem Cell Laboratory, Department of Dermatology, Venereology and Dermato-Oncology, Semmelweis University, Budapest, Hungary; 4 Department of Infectious Diseases, College of Veterinary Medicine, University of Georgia, Athens, GA USA; 5 Intrinsic Life Sciences, La Jolla, CA USA

**Keywords:** Immunology, Stem cells

## Abstract

Bone marrow stromal cells (BMSCs) have immunomodulatory activities in numerous species and have been used in clinical trials. BMSCs also make antibacterial agents. Since hepcidin is known to have antimicrobial effects in fish, we wondered if it might also be used as an antimicrobial agent by mammalian BMSCs. In the present study, we show hepcidin expression in both mouse (mBMSC) and human BMSCs (hBMSC). We observed a hBMSC hepcidin-dependent degradation of ferroportin in HEK-293 reporter cells in vitro. In human and mouse bone marrows (BM) we detected hepcidin-positive BMSCs in close proximity to hematopoietic progenitors. The conditioned culture medium of hBMSCs significantly reduced bacterial proliferation that was partially blocked by a hepcidin-neutralizing antibody. Similarly, medium in which hepcidin-deficient (*Hamp*^*−/−*^) mouse BMSCs had been grown was significantly less effective in reducing bacterial counts than the medium of wild-type cells. In a zymosan-induced peritonitis mouse model we found that mBMSC-derived hepcidin reduced the number of invading polymorphonuclear (PMN) cells in the peritoneal cavity. Our results show that BMSC-derived hepcidin has antimicrobial properties in vitro and also reduces inflammation in vivo. We conclude that hepcidin should be added to the expanding arsenal of agents available to BMSCs to fight infections and inflammation.

## Introduction

Bone marrow-derived stromal cells (BMSCs or bone marrow-derived MSCs) comprise the major non-hematopoietic cell population in the bone marrow (BM). A subpopulation of BMSCs, skeletal mesenchymal stem cells, can differentiate into bone, cartilage and adipose tissue^1^, and also support hematopoiesis within the BM niche being in close proximity to hematopoietic progenitors^2–4^. BMSCs were reported to reduce T lymphocyte proliferation^5^. This observation led to their use as a treatment for graft versus host disease in humans^6^. Their immunomodulatory actions have also been studied in a variety of mouse models such as sepsis^7^, asthma^8^ and myocardial infarction^9^. Secretion of disease-modifying factors—different ones in different problems—seems to be responsible for the effects seen: prostaglandin E2 in sepsis, transforming growth factor-β (TGF-β) in asthma and TNF-(tumor necrosis factor)-α-induced protein 6 (TNAIP-6) in cardiac ischemia^10^.

Even though BMSCs improve the survival of septic mice^7^ by inducing proinflammatory macrophages (M1) to change their character to anti-inflammatory (M2) macrophages, little attention has been paid to the possibility that they could also secrete antibacterial factors themselves as immune cells do. These include LL-37 (cathelicidin)^11^ and cationic antimicrobial peptides^12^, which disrupt the integrity of bacterial membranes. Hepcidin (HAMP), a peptide hormone produced primarily by the liver, is mainly known as a master regulator of iron homeostasis. It lowers plasma iron levels by inhibiting cellular iron efflux through the degradation of the iron exporter, ferroportin, in duodenal cells, macrophages and hepatocytes^13^. Hepcidin has been isolated from plasma^14^, human urine^15^, and fish liver^16^, and is similar to antimicrobial peptides called defensins^14^ that also disrupt the integrity of the bacterial cell membrane. Menstrual blood-derived human BMSCs were reported to express hepcidin mRNA and were shown to be effective in decreasing bacterial growth^17^. It was also shown that mouse BMSCs, after conditioning with staphylococcal enterotoxin B, upregulate hepcidin mRNA expression and enhance bacterial clearance in septic mice^18^. Based on these original studies, subsequent reviews anticipated the general presence of hepcidin in BMSCs as a component of their antimicrobial peptide arsenal^19–21^.

Since in the bone marrow niche leptin receptor and nestin-positive peri-sinusoidal and peri-arteriolar BMSCs neighbor and support hematopoietic stem and progenitor cells (HSPCs)^3,22^, we hypothesized that BMSCs might also make and release hepcidin locally to add it to their arsenal of antibacterial agents. If so, hepcidin could help protect hematopoietic cells from microbial pathogens. It could also contribute to the beneficial effects of BMSCs given intravenously in sepsis.

## Methods

### Human tissues and primary cells

Cryopreserved, clinical-grade adult human MSCs aspirated from the iliac crest of healthy donors were obtained from the Bone Marrow Stromal Cell Transplantation Center of the National Institutes of Health (NIH), Bethesda, MD, USA. Informed consent was obtained from all the participating individuals. Research was conducted according to the principles of the Declaration of Helsinki. Research protocols (10-CC-0053) were approved by the Institutional Review Board of the NIH (see clinical protocol NCT01071577 at clinicaltrials.gov). The human BMSCs have been fully characterized for clinical use and were positive for CD105, CD73 and CD90 and negative for CD45, CD14, CD34, CD11b markers. The 5 different donor cells used in this work were among the ones that were used for detailed studies on subject variability and replicative senescence in a study published by the bone marrow transplantation department^23^ at NIH.

Human liver tissue was obtained post-mortem from an anonymous, non-fetus, donor in the 1980’s and donated to the NIH research team. No proof of donor consent was required to obtain this tissue from an anonymous donor.

### Animals

All animal studies were approved by the NIDCR/NIH/DHHS Animal Care & Use Committee. All animal studies were performed according to relevant guidelines and regulations and to approved institutional protocol (NIDCR ASP # 13-714) as described earlier^24^. All methods used in the mouse studies are reported in accordance with ARRIVE guidelines. The hepcidin-deficient (*Hamp*^*−/−*^) transgenic mice were a generous gift from Dr. Tomas Ganz at UCLA.

### BMSC isolation

BMSCs were cultured from mouse bone marrow as described previously^25^, with some modifications. We have modified the BMSC separation protocol published earlier, by performing the immunomagnetic separation of BMSCs before using them in the in vivo experiments. To achieve this, we added rat anti-mouse lineage antibodies (anti-CD45, anti-CD11b) to mouse bone marrow cell suspension after ACK lysis, and the cells were depleted using anti-rat magnetic beads (Biomag, goat anti-rat IgG, Qiagen) as per the manufacturer's instructions. In the original article referred to throughout the manuscript, Miltenyi antibodies were used with a MACS LD separation column. We believe this modification simplified the negative enrichment process and did not change the functional parameters of the final mouse BMSC product.

### PCR

Total RNA was isolated using Agilent (Wilmington, DE) and Stratagene “Absolutely RNA” Microprep kits (Stratagene, La Jolla, CA) according to the manufacturer`s specifications. For some experiments, on-column DNA digestion was performed using RNase-Free DNase Set (Qiagen, Valencia, CA) at room temperature for 30 min. 1 µg of total RNA was reverse transcribed with oligo (dT) primers using Promega’s M-MLV RT kit (Promega, Madison, WI). The resultant cDNA was amplified using the QuantiTect SYBR Green RT-PCR kit (Qiagen). RT-PCR conditions were as follows: 95 °C for 15 min initial activation of the Taq polymerase and denaturation of the reverse transcriptase and then 45 cycles of 94 °C for 15 s denaturation, 60 °C for 30 s annealing, and 72 °C for 30 s extension. Quantitative PCR was done using TaqMan primer assays for human hepcidin from Applied Biosystems (Foster City, CA; see Supplementary Table 1) and with the TaqMan Gene Expression Master Mix (Applied Biosystems). Data were normalized to beta actin expression and the hepcidin expression of control samples.

The PCR products were run on 2% agarose gels and visualized using ethidium bromide.

### Bacteria

*Escherichia coli* (E. coli) and *Pseudomonas aeruginosa* (P. aeruginosa) PAO1 were purchased from The American Type Culture Collection (ATCC, Manassas, VA, USA). Fresh bacterial cultures were prepared for every experiment from frozen aliquots. Bacteria were cultured overnight in 3 mL of Lysogeny broth (LB) medium, washed twice, and resuspended in sterile PBS. Bacterial concentrations were adjusted to 10^9^/ml after measuring the optical density of the bacterial suspensions using a spectrophotometer.

To determine changes in HAMP mRNA levels after exposing BMSCs to *Pseudomonas aeruginosa*, TNF-α or INF-γ. 400,000 hBMSCs were plated in 6-well plates overnight with or without penicillin and streptomycin, followed by incubation in 10 ng/mL TNF-α or 10 ng/mL INF-γ for 6 h, or live *Pseudomonas aeruginosa* (0.1 *P. aeruginosa*: BMSC multiplicity of infection, 40,000 bacteria per well) for 4–9 h. Bacterial exposure of hBMSCs did not affect their viability or confluency as assessed by light microscopy and Trypan Blue dye exclusion assay (data not shown). Following these challenges, the BMSCs were washed in PBS and lysed in Stratagene lysis buffer. Then RNA and cDNA were prepared, and PCR was run (see above).

### Immunofluorescence

Human BMSCs were seeded in 8-well chamber slides (Thermo Scientific, Rockville, MD) at a density of 5000 cells/well (8 well chamber slide surface area: 0.8 cm^2^/well) and allowed to attach to the surface overnight in DMEM. After washing the cells with PBS, they were fixed for 10 min at room temperature with 4% PFA / PBS, quickly washed in 0.1 M Tris (pH 8.0) to remove salts, and air-dried. Power Block Universal Blocking Reagent (Biogenex, San Ramon, CA) was used to block non-specific binding sites, and then incubated with the primary antibody for 24–48 h at 4 °C. After blocking endogenous peroxidase with Peroxidase Block (DAKO, Carpinteria, CA), secondary antibodies and tyramide-fluorophores were used to detect the target protein^26^. Cell nuclei were stained using DAPI. See Supplementary Table 1 for sources and dilutions of the primary and secondary antibodies, and other reagents used for immunostaining. The sections were analyzed using a Leica DMI6000 inverted fluorescent microscope and LAX (Leica Camera Inc, Teaneck, NJ) software, sometimes with Z series and deconvolution. Immunohistochemical controls included no primary antibody (hBMSCs) or the use of Hamp knockout cells (mouse).

Mouse bone marrow sections were cut in a cryostat following decalcification of femurs from Nestin-GFP mice. In the bone marrow GFP/Nestin in these mice labels BMSCs that support hematopoiesis. Hepcidin was then visualized using a specific antibody (Abcam 81289) at 1:100 at 4 °C overnight followed by an anti-rabbit Alexa 594 (1:1000). Finally, hematopoietic stem cells were labelled using an antibody to c-kit (BAF1356, R&D) at 1:500, O/N at 4 °C, and developed the staining with a secondary antibody conjugated to far red fluorochrome (Alexa-647) at 1:1000. The sections were imaged with a Leica DMI6000 inverted fluorescence microscope. A Z stacks were taken at 0.5 µm intervals and the image was deconvoluted by the LAX software.

Human bone marrow sections (purchased from US Biomax Inc., HuFPT240) were deparaffinized and incubated with a 1:2000 dilution of a CD34 antibody (Abcam 81289), followed by an anti-rabbit polymer HRP and amplified using an Opal-650 tyramide (Perkin-Elmer) at 1:200 dilution. After heat removal of the rabbit primary antibody, the hepcidin antibody (Abcam 30760) was placed on the sections at 1:300 dilution at 4 °C O/N, followed by an Alexa-594 secondary antibody at 1:1000. Finally, the leptin receptor antibody was used (BAF-497 R&D) at a 1:100 dilution at 4 °C O/N then visualized with Alexa-488 at 1:1000 for 1 h at RT. Spectral imaging was performed on a Nikon A1R + confocal microscope equipped with an A1-DUS 32 channel spectral detector. Images were acquired using a CFI Plan Fluor 40 × 1.3 NA objective and spectral unmixing performed against library spectra of AF488, Rhodamine Red X, and AF647.

### Cell lines, tissue culture and hepcidin response experiment

HEK 293 cells were cultured in DMEM containing 10% fetal bovine serum and human BMSCs were cultured in alpha MEM 10% FBS and IL-6 10 ng/ml. Both cell lines were supplemented with Antibiotic Antimycotic Solution (A-5955, Sigma-Aldrich, St. Louis, MI), and grown at 5% CO_2_ at 37 °C. For co-culturing the two types of cells we used a 1 to 1 mix of their individually optimized medium.

Human ferroportin-GFP (hFpn-GFP) cells were generously donated by Elizabeta Nemeth, UCLA^13^. These cells are HEK293T cells stably infected with a doxycycline-inducible lentiviral construct encoding human ferroportin with a C-terminal GFP tag. hFpn-GFP cells were selected and maintained with 0.5 µg/mL puromycin. GFP induction occurred with the addition of doxycycline 100 ng/mL 24 h prior to establishing co-cultures.

Co-culture: hBMSCs (40,000/well) were plated in 250 µL medium in a 24-well poly-l-lysine coated plate with glass coverslips one day prior to the addition of the hFpn-GFP cells. The following day, 10,000 doxycycline induced hFpn-GFP cells in 250 µL were added to each well. In one set of experiment an anti-hepcidin antibody (Mab583, 0.22 µg/µL) was also added to the wells.

Supernatant (SN): hBMSCs (40,000/well) were plated in 250 µL in a 24 well plate and stimulated by IL-6 overnight. 10,000 doxycycline induced hFpn-GFP cells in 250 µL were plated on poly-l-lysine coated 24 well plate with glass coverslips and grown overnight. The following day, the SN from the IL-6 stimulated BMSCs were added to each well. In addition to the BMSC SN, in one set of experiment the anti-hepcidin antibody (Mab583, 0.22 µg/µL) was also added. All conditions were done in triplicates. Cells were washed with PBS and fixed 36 h later with 4% PFA. Slides were mounted with ProLong antifade.

To demonstrate that the reporter hFPT-GFP staining moved away from the membrane to the area of the reporter HEK-293 T cell nuclei, the relative quantitation of the green fluorescence within this area was done using the Image J (NIH) software. Pictures recorded in RGB (red–green–blue) code were split according to individual color channels and the contours of cell nuclei were determined using the blue channel picture (DAPI staining, n = 8 nuclei/picture). These contours were then copied to the green channel image and the mean green fluorescent intensity was determined within the contour lines. These intensity values were normalized by dividing them with the area value of the cell nuclei.

### Hepcidin dot-blot assay

Livers were removed from freshly euthanized wildtype (C57BL/6) and one Hamp^−/−^ mouse. The livers were lysed in RIPA buffer with the addition of phosphatase and proteinase inhibitor cocktail (ThermoScientific, Rockford, IL). Lysates were homogenized on ice and centrifuged to pellet debris. Protein was quantified using the DC protein Assay, (Bio-Rad, Hercules, California). Transfer membrane Immobilon-P^SQ^ (Millipore, Burlington, MA) was pretreated, and then 60 µg of each lysate was dotted onto the blot into the circles drawn on the membrane. The membrane was allowed to dry overnight and reactivated. The membrane was blocked in 5% milk for 1 h and incubated in Anti-Hepcidin-25 antibody (ab30760, Abcam) overnight at 4 degrees. The membrane was washed and incubated in Goat Anti-Rabbit IgG-HRP, (SouthernBiotech, Birmingham, AL) for one-hour at room temperature. The membrane was washed and developed with Immobilon ECL Ultra Western HRP Substrate, (Millipore).

### Co-culture experiments (mouse and human)

To a 96-well plate mBMSCs (WT or *Hamp*^*−/−*^) or hBMSCs obtained from normal volunteers following informed consent^27^ were added in varying concentrations (2500, 5000, 10,000 or 25,000 cells/well). After allowing the cells to settle (2–3 h) corresponding monocytes and macrophages, were added to the wells, murine (RAW264.7 and J774.2) or human (THP-1) at 100,000 cells/well. All cells were cultured in phenol-free media and 3% FBS. After overnight incubation 1 µg/mL of either LPS or zymosan was added to the co-culture to mimic inflammation. The SN from the wells was collected 6 h post treatment for the mouse co-cultures and at 6 h and 24 h for the human co-cultures from separate plates. The plates were briefly spun (100×*g* 1 min) prior to SN removal to prevent macrophages in the SN. The SNs were not filtered or altered prior to the assay, and control wells containing only phenol-free media and 3% FBS were included to account for any counts from the FBS. Levels of TNF-α and IL-10 in the SN were measured in quadruplicates using DuoSet ELISA kits (R&D Systems) according to the manufacturer`s instructions.

### Bacterial proliferation studies

The BMSC antimicrobial properties were studied by an in vitro* E. coli* proliferation assay as described earlier^28^ using conditioned medium from human or mouse BMSCs (comparing WT vs. Hamp KO). Filtered BMSC supernatants were added to *E. coli* suspended in LB medium in a 1:1 ratio, aliquoted in 96-well microplates, and cultured for 16 h. Bacterial growth was assessed by measuring optical density at 600 nm. In studies of human BMSCs, mouse monoclonal and rabbit polyclonal anti-hepcidin antibodies (affinity purified) were used to block the action of hepcidin. Mouse and a rabbit IgGs (Jackson Chrompure: ammonium sulfate precipitation plus gel filtration) served as isotype controls in all experiments. In some experiments using human BMSCs, hepcidin expression was boosted by the addition of 10 ng/mL human IL-6 for 9 h. Supernatants without washing were removed and exposed to *E. coli* to measure bacterial growth. Control samples (without BMSCs) showed that the presence of IL-6 did not have any effect on *E. coli* growth.

### Peritonitis mouse model

For all experiments, 6- to 24-week-old *Hamp*^*−/−*^ and age- and gender-matched C57BL/6 WT control mice were used. 400 µg of zymosan in 800 µl of PBS were injected intraperitoneally (ip) to induce inflammation. 15 min after inducing inflammation, 0.5–1.0 × 10^6^ WT or *Hamp*^*−/−*^ CD45- and CD11b-depleted, passage 3–5 BMSCs were injected i.p. in 200 µL PBS. At 18 h, mice were anesthetized with pharmaceutical-grade isoflurane and peritoneal lavages were performed with 5 mL of ice-cold PBS for ELISA, cell counting and flow cytometry analysis. We stained the peritoneal lavage cells with DAPI, CD45-APC, CD11b PerCPCy5.5, and Gr1-PE antibodies, and calculated the percentage of the Gr1 + CD11b + polymorphonuclear lymphocytes (gating strategy is presented in Supplementary Fig. 1). For specific antibodies and reagents, see Supplementary Table 1. At the end of the experiments, mice were euthanized by CO_2_ asphyxia followed by cervical dislocation to confirm death.

### Enzyme linked lectin assay (ELLA)

An ELLA assay was used to quantify human hepcidin concentrations in BMSC SNs (Bio-Techne, Minneapolis, MN). Standard growth media of Hu BMSCs grown to 90% confluency was replaced with 8 mL of phenol free media supplemented with 3% FBS and antibiotics. Cells were stimulated with BMP2 (200 ng/mL), IL-6 (50 ng/mL) and LPS (2 μg/mL) for 24 h. The SNs of each plate were removed and placed in a Vivaspin-15R (Sartorius Corporation, Bohemia, NY) to concentrate the media. The tubes were spun at 2500×*g* for 1 h 25 min. Cell lysates were lysed in RIPA buffer with the addition of phosphatase and proteinase inhibitor cocktail (ThermoScientific, Rockford, IL). Lysates were homogenized on ice and centrifuged to pellet debris. SNs and lysates were used to measure the concentration of hepcidin using the Protein Sample Ella-Simple ELISA System (Revision 3.5.2). Hepcidin was supplied by ProteinSimple and concentrated, untreated media were included as controls.

### Statistical analysis

Statistical analyses were performed using t-tests or one-way ANOVA, depending on the number of samples in the experiments. For fluorescence image quantitative analysis, Friedman test and Dunn’s multiple comparisons test was utilized. Statistically significant changes: p < 0.05.

## Results

### BMSCs from humans and mice express hepcidin

**Figure 1:**
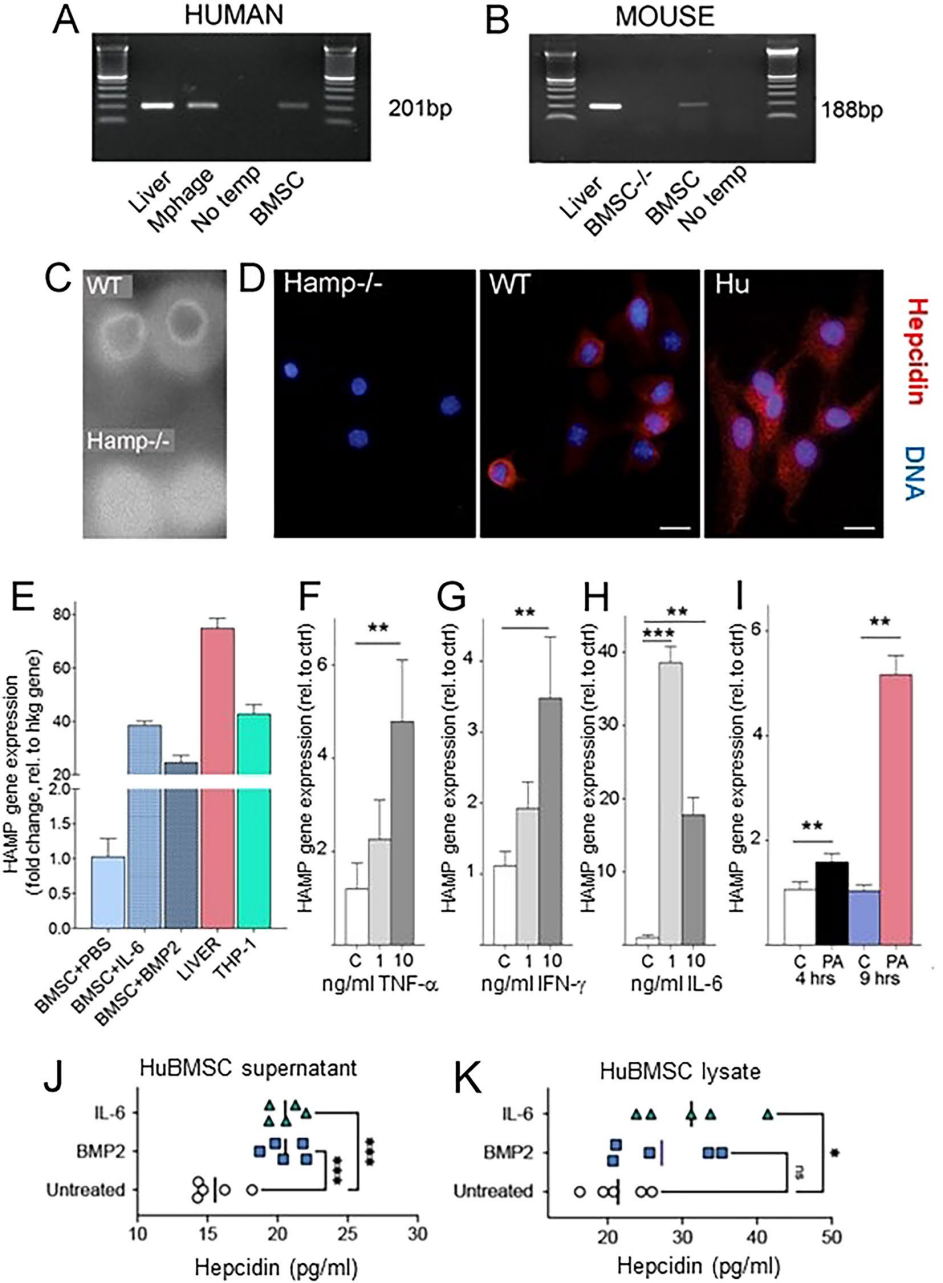
Hepcidin is expressed in BMSCs and is inducible upon microbial and inflammatory stimuli. (**A**) PCR showing human hepcidin gene (*HAMP*) expression in BM stromal cells (BMSCs). *HAMP* primer product size is 201 bp. (**B**) PCR showing mouse hepcidin gene (*Hamp*) expression. *Hamp* primer product size is 188 bp. (**C**) Dot blot of wild type and *Hamp*^*−/−*^ mice livers to show specificity of the antibody used. (**D**) Immunofluorescent detection of hepcidin in BMSC isolated from *Hamp*^*−/−*^ and WT mice or obtained from human volunteers (nuclear staining in blue and hepcidin in red). Scale bars represent 10 µm (mouse) and 16 μm (human). (**E**) Hepcidin mRNA expression in unstimulated hBMSCs, IL-6- and BMP2-stimulated hBMSCs, human liver and THP-1 macrophage cells [compared to housekeeping (hkg) control gene expression] (**F**–**H**): Human BMSC *HAMP* expression in response to 6 h (**F**) TNF-α, (**G**) INF-γ and (**H**) IL-6 stimulation at different doses [“c” (control, no cytokine), 1 ng/mL and 10 ng/mL]. (**I**) *HAMP* mRNA expression in control hBMSCs or hBMSCs stimulated with *Pseudomonas aeruginosa* for 4 or 9 h. Human hepcidin Taqman qPCR results were normalized to beta-actin expression. (**J**, **K**) Human BMSC cultures from five independent donors were left unstimulated or were stimulated with 200 ng/mL BMP2 or 50 ng/mL IL-6 for 24 h in vitro. Hepcidin concentrations were determined by ELLA in (**J**) collected and concentrated SNs and (**K**) cell lysates. One-way ANOVA, *p < 0.05; **p < 0.01; ***p < 0.001.

Hepcidin mRNA was detected in cultured BMSCs by means of RT-PCR in both human and mouse BMSCs as shown in (Fig. 1A) and (Fig. 1B), respectively. Human liver and a human monocytic/macrophage cell line (THP-1) served as positive controls (Fig. 1A). In mice, liver was used as the positive control and BMSCs derived from the *Hamp*^*−/−*^ mice as a negative control (Fig. 1B). Due to the small size of the hepcidin molecule, we were not able to perform Western blotting. Using a dot-blot assay, however, the antibody used was able to bind to the protein isolated from the BMSCs from WT, but not from *Hamp*^*−/−*^ mice (Fig. 1C). Using this antibody, hepcidin could be visualized by immunofluorescence in murine and human BMSCs (Fig. 1D). Mouse BMSCs were prepared from WT or *Hamp*^*−/−*^^29,30^ mouse long bones. Human BMSCs were obtained from healthy volunteers and expanded in culture. We designed a functional assay to confirm production and release of hepcidin from cultured human BMSCs. A comparison of *Hamp* mRNA expression among different tissues and stimulated hBMSCs is shown in Fig. 1E.

### Microbial and inflammatory stimuli upregulate hepcidin mRNA in human BMSC

To address whether bacterial challenge upregulates the expression of *HAMP* in hBMSCs, huBMSCs were stimulated in vitro with increasing concentrations of the proinflammatory cytokines, TNF-α (Fig. 1F) and IFN-γ (Fig. 1G) overnight. A dose-dependent and significant (p < 0.01) increase in *HAMP* mRNA levels was observed in human BMSCs. IL-6 stimulation of cultured hBMSCs also resulted in a significant increase of *HAMP* mRNA (Fig. 1H) as was reported in cultured liver cells^13^. Since BMSCs have been shown to exert antimicrobial functions against *Pseudomonas aeruginosa*^31–33^, live *P. aeruginosa* was added to human BMSC cultures and observed an increase in *HAMP* mRNA expression after 4 h that became even greater after 9 h (Fig. 1I). Lysates and SNs of hBMSCs contained detectable amounts of hepcidin (Fig. 1J,K). When hBMSCs were stimulated with BMP2 or IL-6, significant increases were observed in hepcidin levels, both in SNs (Fig. 1J) and in cell lysates (Fig. 1K).

### Human BMSCs release hepcidin in vitro

The doxycycline-inducible GFP reporter HEK293 cell line (referred to as hFpn-GFP cells) was used to detect hepcidin production in hBMSCs since there are no commercially available ELISA assays available^34^. hFpn-GFP cells express GFP when ferroportin (FPT), the molecule responsible for iron export from cells, is expressed^35^. The cells were studied in two different conditions.

**Figure 2:**
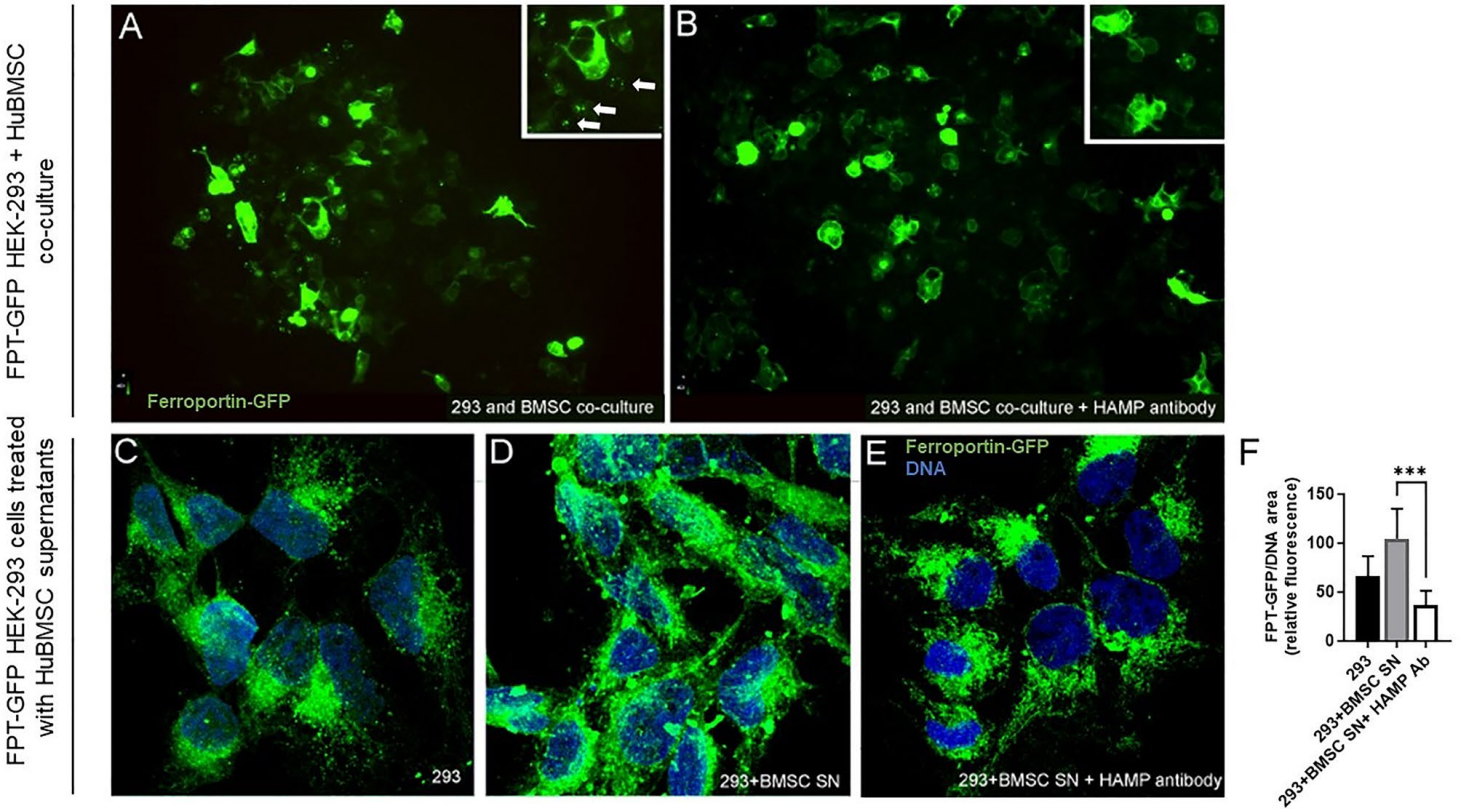
Ferroportin degradation is promoted by BMSC-derived hepcidin in vitro. (**A**) Human BMSCs were co-cultured with the reporter cell line where ferroportin (the molecule that is responsible for transporting iron from the cells) is tagged with doxycycline-inducible GFP. The ferroportin staining shows a punctate pattern as it is being broken down due to the presence of hepcidin (marked by white arrows). (**B**) When hepcidin antibody is added to neutralize the effect the dotty pattern disappears and a general membrane staining presents, suggesting the presence of intact FPT in the cells. (**C**) cultured HEK 293 cells show cytoplasmic staining of FPT-GFP. (**D**) When SN taken from human BMSCs (previously primed with IL-6) is added to the medium, the cells exhibit a granular, much stronger, cytoplasmic and membrane staining pattern (staining is also visible over the nuclei) that (**E**) changes back to a cytoplasmic staining (similar to **C**), after a neutralizing hepcidin antibody is added. (**F**) The amount of cell nucleus-associated FPT is determined by calculating the ratio of cell nucleus-associated mean green fluorescence intensity values and the cell nucleus area indicated by the blue DNA staining. Eight cells were analyzed in each of the three conditions represented in panels (**D**, **E**, **F**). Friedman test and Dunn’s multiple comparisons test. ***p < 0.001. *Ab* antibody, *FPT* ferroportin, *GFP* green fluorescent protein, *ns* non-significant, *SN* supernatant.

In co-cultures the presence of BMSCs induced a punctate staining pattern in hFpn-GFP cells and a decrease in the intensity of membrane GFP staining, suggesting the degradation of FPT (Fig. 2A). When hepcidin-neutralizing antibody was added to the co-culture, much less punctate staining was observed and membrane staining in the reporter cells became more visible (Fig. 2B) suggesting the presence of more intact FPT. In another set of experiments hBMSCs were prestimulated with IL-6 to induce hepcidin production^13^. hFpn-GFP cells show a cytoplasmic expression of FPT (Fig. 2C). When SN from IL-6 stimulated BMSCs was added to the reporter cells, FPT moved to the membrane (Fig. 2D,F), while adding hepcidin-neutralizing antibody appeared to prevent this (Fig. 2E,F). GFP in these cells was mostly membrane-bound with some cytoplasmic distribution (Fig. 2C). These results suggest that FPT translocation and degradation in HEK-293 cells was dependent on BMSC-derived hepcidin.

### BMSC-derived hepcidin reduces bacterial growth in vitro

**Figure 3:**
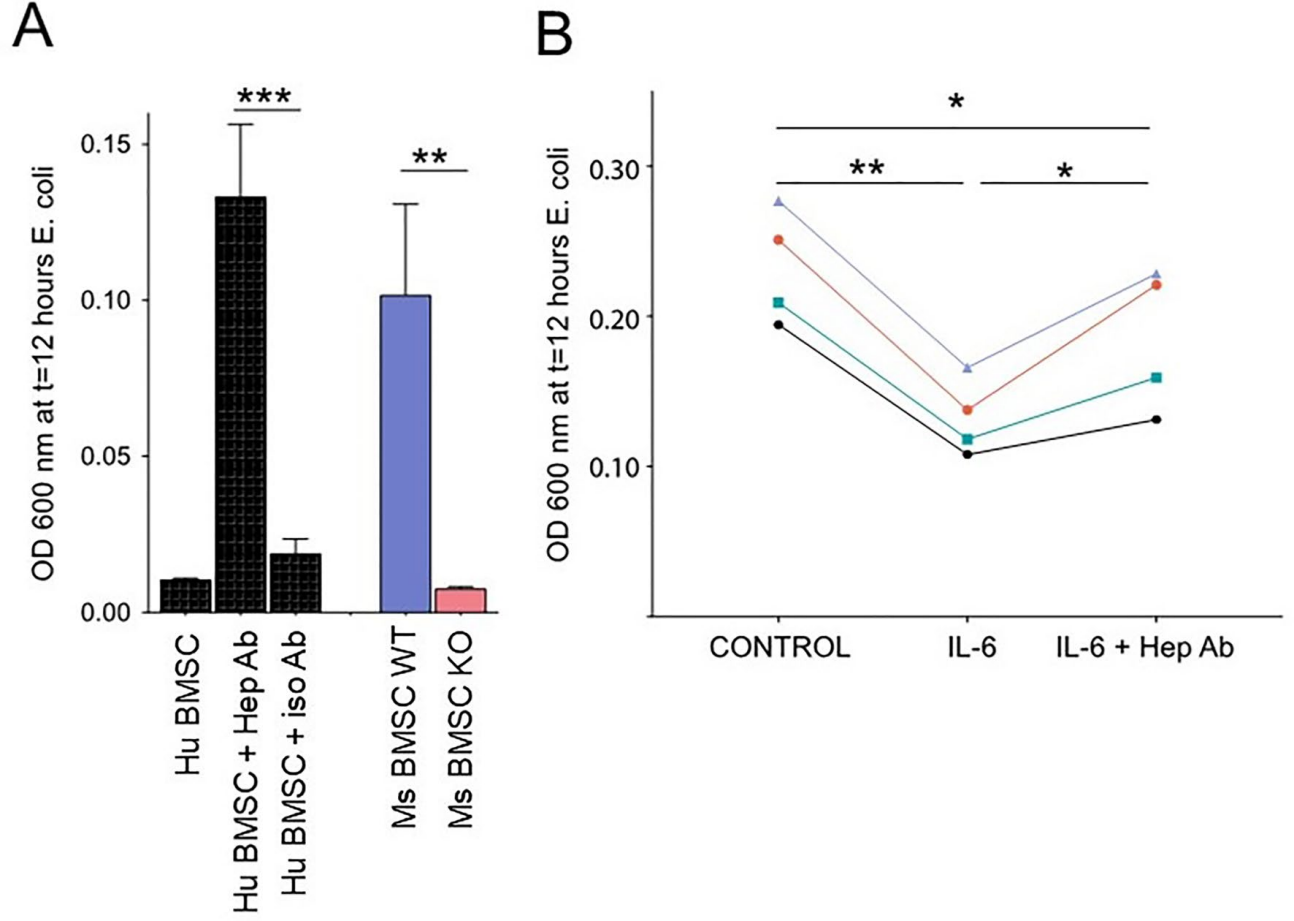
In vitro killing of *E. coli* by human or murine BMSC SNs is hepcidin-dependent. (**A**) Sterile filtered SNs were added to *E. coli* suspension and bacterial cell proliferation was followed by a microplate reader (600 nm) where one scale on the optical density (OD) axis reflects 10^9^/mL *E. coli* and the OD is directly proportional to the bacterial concentration during the measurements. For human cells, polyclonal rabbit anti-hepcidin antibody or isotype control rabbit IgG was added. SN taken from BMSCs cultured from WT mice was significantly more efficient in inhibiting bacterial growth than SN of BMSCs cultured from *Hamp*^*−/−*^ mice. (**B**) SN were taken off and sterile-filtered from cultured control or IL-6-stimulated human BMSCs. There was a significant decrease in *E. coli* proliferation when the BMSCs had been treated with 10 ng/mL IL-6 for 9 h. When hepcidin antibody was added simultaneously to the IL-6 stimulated SN, the decrease in OD was partially blocked, suggesting that hepcidin is at least partially responsible for the anti-proliferative effect of human BMSCs on *E. coli.* *p < 0.05; **p < 0.01; ***p < 0.001.

SNs taken from BMSCs were added to *E. coli* suspension and bacterial cell proliferation was followed for 12 h using a microplate reader (600 nm) by determining optical density (OD) which is directly proportional to the bacterial concentration during the measurements. SN of human BMSCs decreased the proliferation of *E. coli*, which decrease was partially blocked by a hepcidin-specific antibody, but not by the isotype IgG (Fig. 3A) (first three columns). In the same assay, SN from cultured BMSCs derived from WT mice were significantly more efficient in inhibiting bacterial growth than SN of BMSCs from Hamp^−/−^mice (Fig. 3A) (last two columns). In a separate set of experiments, IL-6 was used to induce hepcidin production. Following IL-6 stimulation of cultured hBMSCs, the SN was removed, sterile filtered and tested in the bacterial growth assay as before. The results of four independent experiments show that there was a significant decrease in *E. coli* proliferation when the SN from IL-6-stimulated BMSCs were used. This inhibition of *E. coli* growth was partially blocked when hepcidin antibody was added to the IL-6-stimulated BMSC SN. However, when the isotype-specific, control antibody was used, the growth-inhibitory effect remained unchanged, suggesting that hepcidin is partially responsible for the bacterial growth-inhibitory effect of IL-6-activated BMSC SNs (Fig. 3B).

### BMSC-derived hepcidin affects macrophage cytokine production in both mouse and human

**Figure 4:**
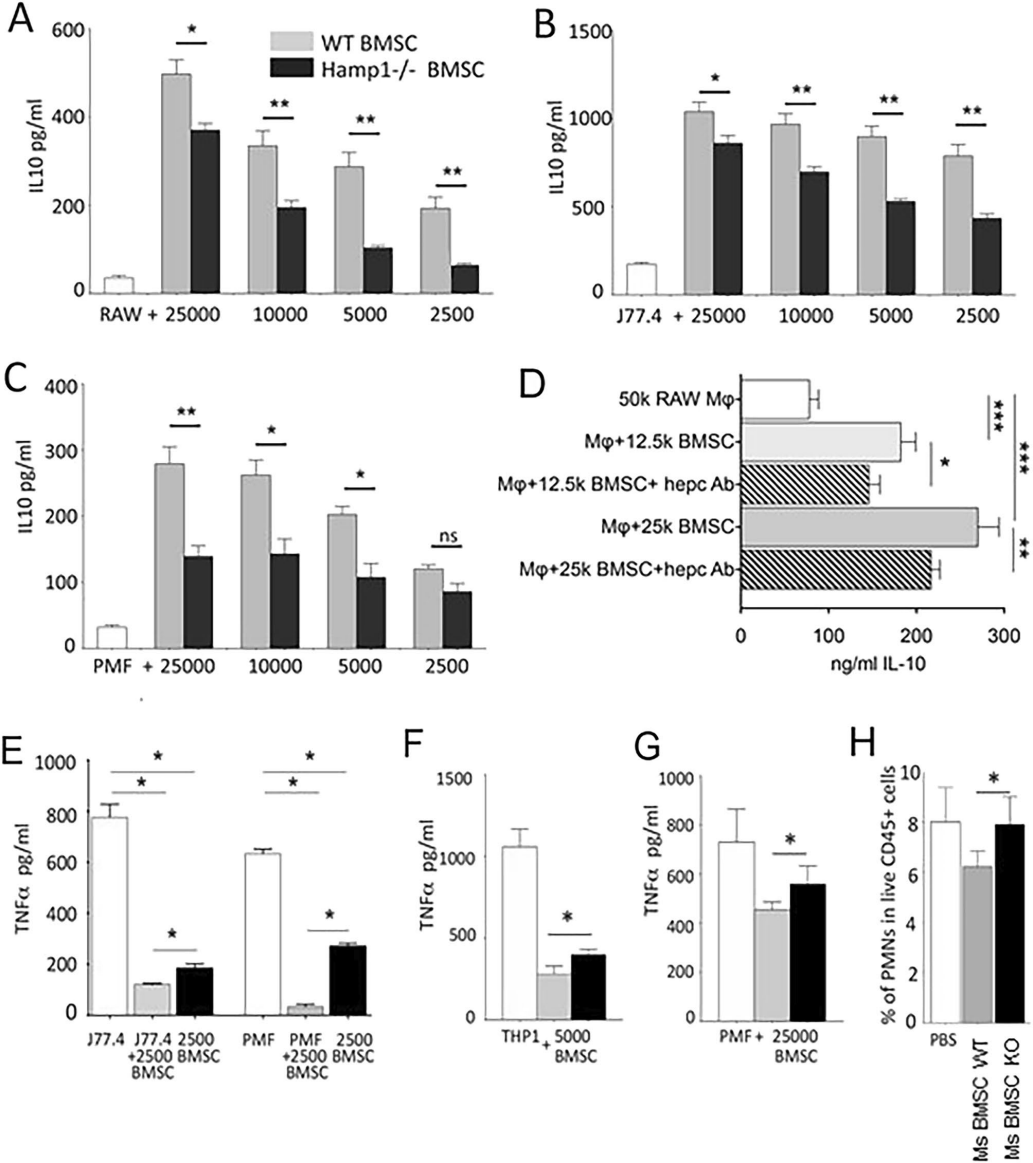
The immunomodulatory effect of mouse BMSCs on macrophages is dependent on hepcidin. IL-10 (**A**–**D**) and TNF-α (**E**–**G**) levels in supernatants of wild-type (WT) or *Hamp*^*−/−*^ BMSCs co-cultured with macrophage cell lines or peritoneal lavage cells after overnight incubation followed by 6 h of LPS stimulation. IL-10 ELISA measurements from SNs of co-cultures of different types of macrophages with WT and *Hamp*^*−/−*^ BMSCs. (**A**) Co-culture of RAW264.7 cells and (**B**) J774.2 cells with BMSCs derived from either WT or *Hamp*^*−/−*^ mice stimulated with 1 µg/mL LPS. (**C**) Co-culture of peritoneal lavage cells with BMSCs. (1 µg/mL LPS). (**D**) Co-culture using RAW264.7 macrophages, wild type BMSCs and anti-hepcidin antibodies (1 µg/mL LPS). (**E**) Co-culture of wild-type or *Hamp*^*−/−*^ BMSCs and J774.2 macrophages (1 µg/mL LPS) or peritoneal lavage cells. (**F**) Co-culture of wild-type or *Hamp*^*−/−*^ BMSCs and human THP-1 cells (1 µg/mL LPS). (**G**) TNF-α levels derived from peritoneal macrophages from zymosan (1 µg/mL)-induced peritonitis. (**H**) Peritoneal cells were stained with CD45, Gr-1 and Cd11b antibodies to detect polymorphonuclear cells (PMNs). As shown we observed a decrease (p < 0.05) in the percentage of PMNs in the live CD45 + population from mice treated with WT BMSCs (gray bar) compared to *Hamp*^*−/−*^ BMSC-treated animals (black bar) or vehicle (PBS—white bar) treated controls. *PMF* peritoneal macrophage. We found the same differences in the complete peritoneal lavage cell numbers between groups (n = 8 per group, pooled data). One-way ANOVA was used. *p < 0.05; **p < 0.01; ***p < 0.001; *ns* non-significant. 5–8 mice were used per group; the experiments were done at least twice.

It has been shown that BMSCs can change the inflammatory character of macrophages to anti-inflammatory^7^ through a PGE2 pathway. We wondered if hepcidin might also modify inflammatory processes and contribute to this effect. Following LPS stimulation of co-cultures of BMSCs and macrophages, IL-10 in the culture medium was measured to reveal how WT vs *Hamp*^*−/−*^ BMSCs effect macrophages obtained from different sources. WT BMSCs induced immortalized mouse macrophages [cell lines, RAW264.7 (Fig. 4A) and J774.2 (Fig. 4B)] as well as peritoneal lavage-derived primary macrophages (PMF) (Fig. 4C) to produce more IL-10 than *Hamp*^*−/−*^ BMSCs do. This effect is cell number-dependent and can be partially blocked by hepcidin-neutralizing antibodies (Fig. 4D) suggesting that a part of (but not the whole) the effect is dependent on hepcidin. In addition, in co-cultures WT BMSCs suppress TNFα production more than *Hamp*^*−/−*^ BMSCs in mouse J774.2 cells (Fig. 4E) and in co-cultures with human THP-1 cells (Fig. 4F). A similar effect is observed when using peritoneal macrophages isolated from mice following zymosan-induced peritonitis (Fig. 4G). Thus, at the level of the macrophage, hepcidin seems to contribute to the anti-inflammatory effects of BMSCs. This may contribute to the action of bolus injected BMSCs in sepsis, where proinflammatory macrophages change character to anti-inflammatory as a result of getting in contact with injected BMSCs^7^.

### Bone marrow stromal cell-derived hepcidin inhibits polymorphonuclear leukocyte recruitment in an in vivo model of peritoneal inflammation

Sterile peritonitis was induced by injecting zymosan into the peritoneal cavity of WT mice. Immediately following the zymosan injection, BMSCs purified from either WT or *Hamp*^*−/−*^ mice were also injected. Eighteen hours later, cells from the peritoneal space were removed by lavage and the numbers of total cells and polymorphonuclear (PMN) leukocytes were determined using FACS analysis. Peritoneal leukocyte and PMN recruitment were significantly (p < 0.05) decreased in mice injected with WT BMSCs compared to animals injected with *Hamp*^*−/−*^ BMSCs or PBS (Fig. 4H) suggesting an inhibitory role of BMSC-derived hepcidin in PMN recruitment.

### Hepcidin-producing cells are in close proximity to hematopoietic stem and progenitor cells (HSPCs) in the BM of mice and human

**Figure 5:**
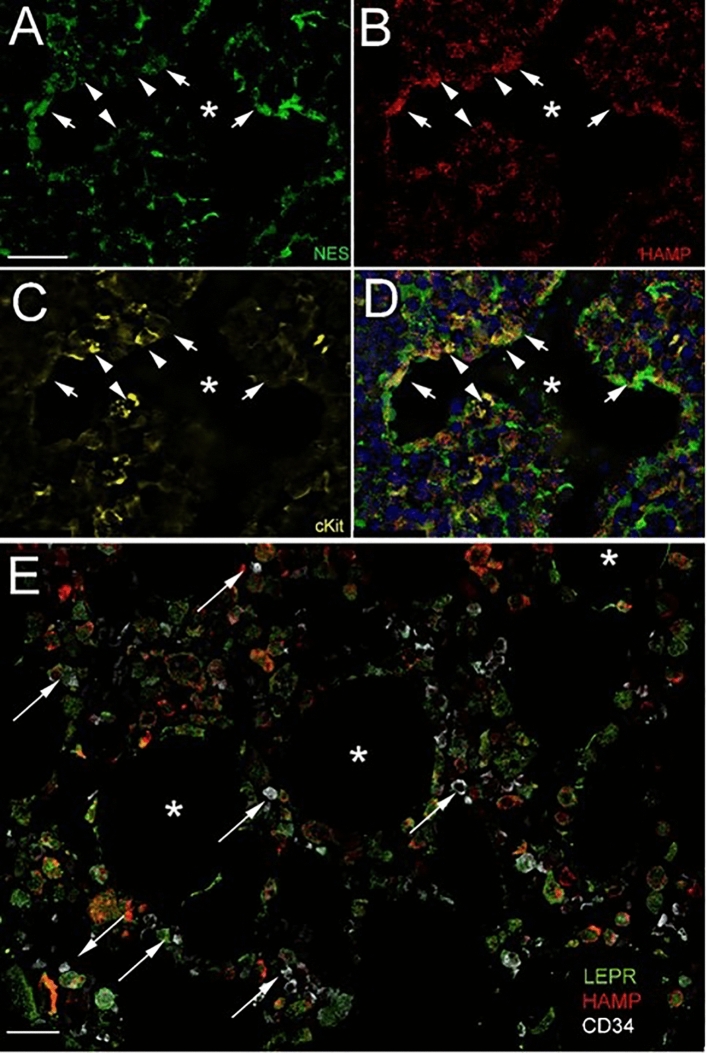
Hepcidin-producing BMSCs are in close proximity to hematopoietic progenitors in both mouse and human BM. Mouse (**A**–**D**) and human (**E**) BM sections are shown demonstrating the close proximity of hepcidin-producing BMSCs to hematopoietic precursors in both species. 16 µm thick sections were cut in a cryostat following decalcification of femurs from Nestin-GFP mice. (**A**) GFP in these mice labels BMSCs that support hematopoesis in the BM. (**B**) Hepcidin was visualized using a specific antibody (Abcam 81289) at 1:100 at 4 °C overnight followed by an anti-rabbit Alexa 594 (1:1000). (**C**) Hematopoietic stem cells (HSCs) were labelled using a biotinylated antibody to c-kit (BAF1356, R&D, 1:500) and developed the staining with a streptavidin-conjugated far red (Alexa-547, 1:1000). The sections were imaged with a Leica DMI6000 inverted fluorescence microscope. A Z-stack was taken at 0.5 µm intervals and the image was deconvoluted by the LAX software. Arrows point at double labelled Nestin/Hepcidin BMSCs, while arrowheads point at c-kit positive HSCs localized next to hepcidin-positive BMSCs. The star points to the lumen of a sinusoid in the BM. (**D**) shows the overlay of the three colors and the cell nuclei are stained with DAPI (blue). (**E**) Similar area from a human BM, where leptin receptor-positive BMSCs (green) also produce hepcidin (red) and are in very close proximity to CD34 -positive hematopoietic stem and progenitor cells (HSPC)s (white-far red, some pointed out by arrows). The lumen of the sinusoids is indicated with stars. This section was imaged by spectral imaging performed on a Nikon A1R + confocal microscope equipped with an A1-DUS 32 channel spectral detector. Scale bars: 50 µm.

We wondered if BMSC-derived hepcidin as an antimicrobial agent might play a role in protecting the BM from pathogens and inflammation. Multiplex immunostaining in BM sections was utilized to look at the presence and topography of hepcidin-producing BMSCs in the intact BM niche. We used Nestin-GFP transgenic mice, where the hematopoietic supportive stromal cells are labelled by endogenous GFP and immunostained for hepcidin and c-kit to label HSPCs (Fig. 5A–D). Nestin is a good marker of perivascular mouse MSCs^36^ and co-localizes with leptin receptor that is expressed on perivascular MSCs around sinusoids^37^. In a section of human BM, the leptin receptor was used as a marker of hematopoietic supportive BMSCs and CD34 to stain for HSPCs. In humans, nestin is not a general marker of MSCs but leptin receptors are expressed in human MSCs^37^. Similar to the finding in mice, CD34-positive hematopoietic progenitors were in close proximity with hepcidin expressing BMSCs in human bone marrow (Fig. 5E).

## Discussion

In the bone marrow, BMSCs play a significant role in regulating the proliferation and differentiation of hematopoietic precursors^2,3,38–40^. It is possible that one important role of the BMSCs is to protect the hematopoietic niche from pathogens, and the production and release of antibacterial peptides could contribute to this. These agents include cathelicidin (LL-37) as its mRNA was found to be 10–20 times more abundant in the human bone marrow than any other tissue examined^41^. Many years later LL-37 was shown to be produced by bone marrow stromal cells^11^. More recently it was discovered that small fragments of LL-37 have high potency to kill a variety of pathogens, such as *Candida albicans*, *Staphylococcus aureus* and *E. coli*^42^.

The present work demonstrates that BMSCs (murine and human) produce hepcidin when exposed to proinflammatory cytokines or Gram-negative bacteria. It was also observed that BMSC-derived hepcidin has antibacterial and anti-inflammatory effects in vitro. The role of iron in infections has long been known and studied^43^ and numerous possible mechanisms of its action were suggested^44,45^. Incorporation of hepcidin into bacterial membranes may cause disintegration of the lipid bilayer and cell rupture. Synthetic human hepcidin was shown to kill bacterial clinical isolates in 30–90 min. Bacterial killing is more rapid at acidic pH^45,46^. Hepcidin also inhibits *M. tuberculosis* growth in vitro^47^. Scindia et al. studied the effect of exogenous hepcidin in kidney in endotoxin- and peritonitis-induced pathologies^48^. They found that exogeneous hepcidin decreased LPS-induced high serum TNF-α levels resulting in decreased glomerular injury. Hepcidin also reduced macrophage IL-6 secretion, and when administered early, it reduced bacteremia in sepsis, thus demonstrating a protective role. Stefanova et al. described that hepcidin deficiency promotes susceptibility to *E. coli* sepsis and demonstrated that hepcidin has a critical role in host defense against *E. coli* infections by clearing non-transferrin-bound iron from the circulation^49^. An in vitro study of murine BM-derived macrophages infected with *Leishmania amazonensis* showed that they upregulate hepcidin in a TLR4-dependent manner, reduce cell surface FPT, and accumulate intracellular iron^50^. Similar results were published with macrophages infected with Chlamydia and Legionella species^51^. This may contribute to the action of bolus-injected BMSCs in sepsis, where proinflammatory macrophages change character to anti-inflammatory because of getting in contact with injected BMSCs^6^.

In the BM niche BMSCs were shown to participate in antigen presentation and were described to recall antigens for *Candida albicans* and Tetanus toxoid. The antigen presentation only occurred when IFN-γ was elevated in the immediate environment of the BMSCs^52^. Another interesting finding suggested that the pro-region of the hepcidin precursor might have a bacteriostatic effect by binding to the bacterial DNA and interfering with its transcription^53^.

Several interesting mechanisms have been suggested in the literature. Interleukin-6 is known to induce hepcidin synthesis through binding to the IL-6R and then activating the JAK/STAT3 pathway that induces the transcription of hepcidin in liver cells^54^. A similar induction is achieved by bone morphogenetic protein 2 and 6 (BMP2, BMP6, respectively) through the SMAD pathway^55–58^. A recent work also suggests that in addition to working together with the IL-6 pathway to induce hepcidin, IL-1β also stimulates the expression of the transcription factor C/EBP that induces hepcidin transcription through binding to the hepcidin promoter^59,60^. Thus, it seems that hepcidin is regulated at many levels by different inflammatory cytokines.

In the zymosan-induced peritonitis model, hepcidin reduced the number of invading immune cells. Intraperitoneally injected BMSCs were demonstrated to form aggregates with immune cells^61^, thus being able to regulate their fate. Nemeth et al.^62^ suggested that hepatocyte-derived hepcidin is an acute-phase II reactant since hepcidin expression was only induced by IL-6, but not by TNF-α or IL-1. In contrast, increased hepcidin expression was observed in BMSCs upon stimulation with TNF-α and with IFN-γ, suggesting that BMSC-derived hepcidin may not be related to the acute phase reaction.

Our present study determined that—in addition to BM resident macrophages—the BM stromal cell population (BMSCs) also produces hepcidin that responds to stimulation by cytokines. In the BM of mice as well as humans the hepcidin-expressing BMSCs are in very close proximity to the hematopoietic stem and progenitor cells (HSPCs). This finding suggests that BMSCs might be able to use hepcidin as an antimicrobial agent to fight any potential pathogen in the immediate environment of the HSPCs. Our findings indicate that BMSC-derived hepcidin has a dual effect: (1) hepcidin can directly reduce the proliferation of pathogens through which mechanism it may protect hematopoietic stem and progenitor cells in the BM niche, and (2) BMSC-derived hepcidin can limit the number of infiltrating polymorphonuclear cells (PMNs) in vivo (in our peritonitis model). This may help to protect BM cells from oxidative damage by invading PMNs as other defense regulatory peptides have been suggested to do^12^.

Future work should address the specific function of hepcidin in the BM niche and the relevance of our findings in a clinical setting.

### Supplementary Information

Supplementary Information.

Supplementary Figure 1.

Supplementary Information 1.

Supplementary Information 2.

Supplementary Table 1.

## Data Availability

The data of this manuscript will be available following publication upon request from the authors. Please contact Dr. Eva Mezey at mezeye@nidcr.nih.gov.
