# Supplementary material for: Bone marrow stromal cell-derived hepcidin has antimicrobial and immunomodulatory activities
Source: Sci Rep. 2024 Feb 17;14:3986. doi: 10.1038/s41598-024-54227-1 (PMC10874407; doi:10.1038/s41598-024-54227-1)
Supplement: Supplementary file 1 — Supplementary Information. [file 41598_2024_54227_MOESM1_ESM.docx]

**Supplementary Figure 1. Flow cytometry gating strategy to determine the percentage of PMNs in peritoneal exudate of mice.**

Mice were injected with 400 µg of zymosan intraperitoneally and given negatively enriched 5x105 WT or Hamp1 KO BMSCs, or 200µL PBS as a cell-free control. After 18 hours, peritoneal lavages were collected for cell counting and flow cytometry analysis (FACS). Peritoneal lavage cells were stained with DAPI, CD45-APC, CD11b PerCPCy5.5, and Gr1 PE antibodies, and the percentage of the Gr1+ CD11b+ polymorphonuclear lymphocytes were calculated among all CD45^+^ cells. Mean+/-S.D. n=3-5.
