# Supplementary figures and images for: Bone marrow stromal cell-derived hepcidin has antimicrobial and immunomodulatory activities
Source: Sci Rep. 2024 Feb 17;14:3986. doi: 10.1038/s41598-024-54227-1 (PMC10874407; doi:10.1038/s41598-024-54227-1)

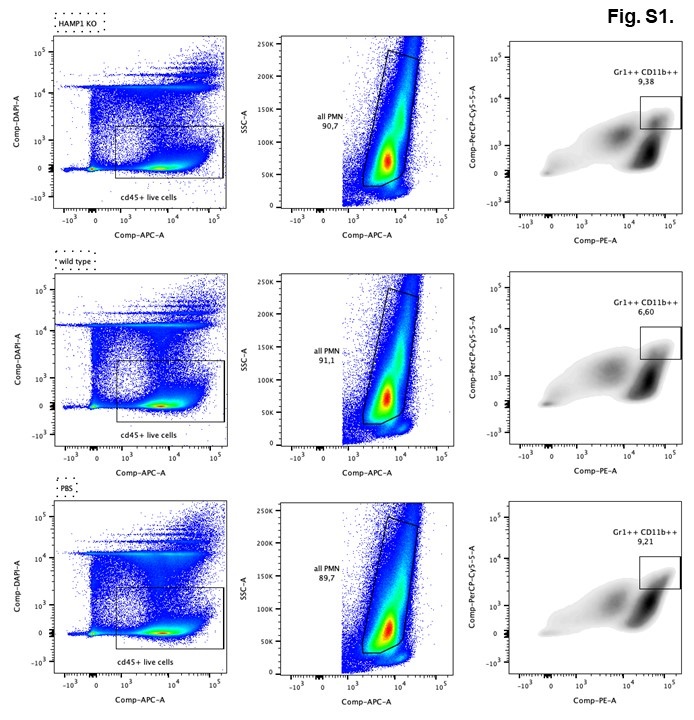

Supplement: Supplementary file 2 — Supplementary Figure 1. [file 41598_2024_54227_MOESM2_ESM.jpg]

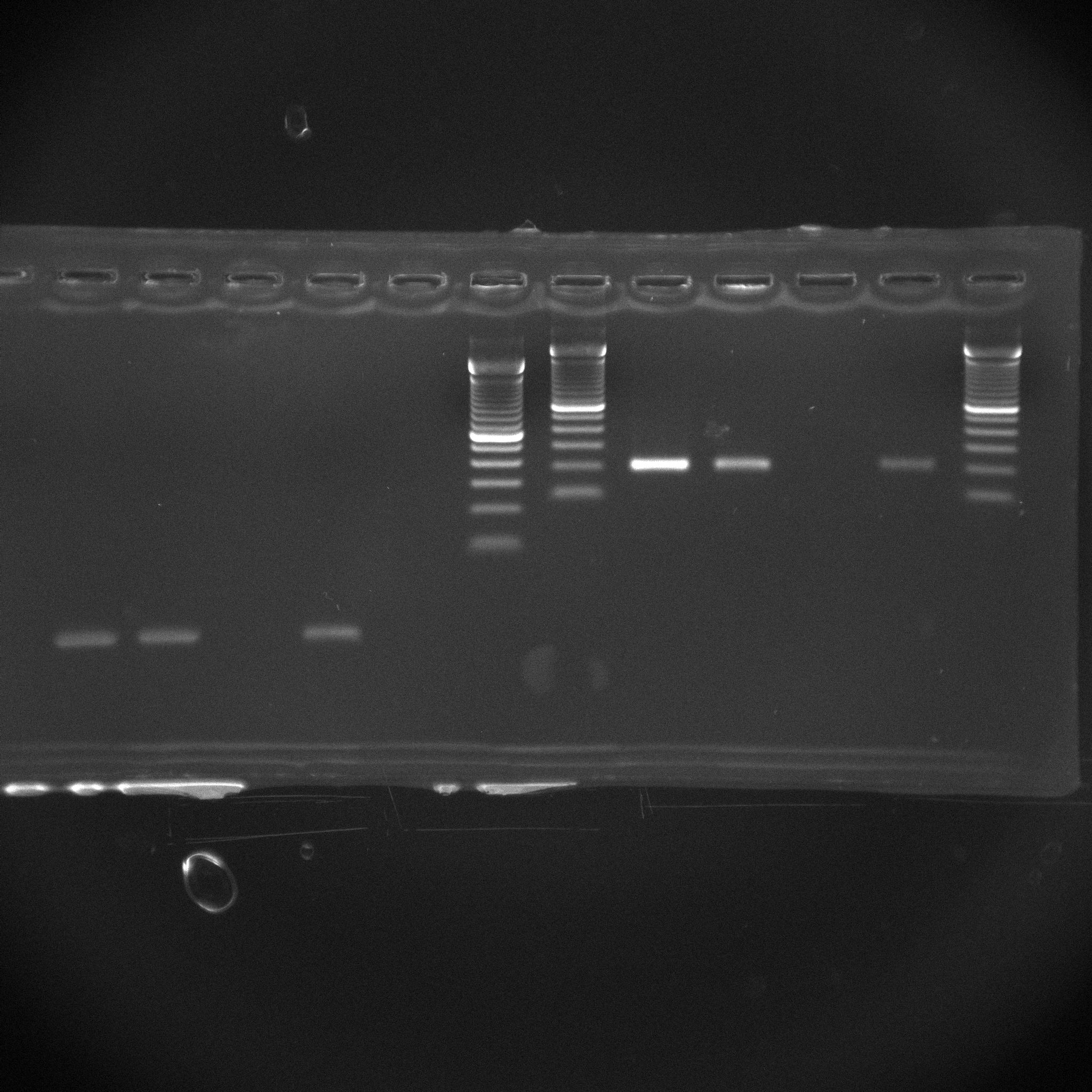

Supplement: Supplementary file 3 — Supplementary Information 1. [file 41598_2024_54227_MOESM3_ESM.tif]

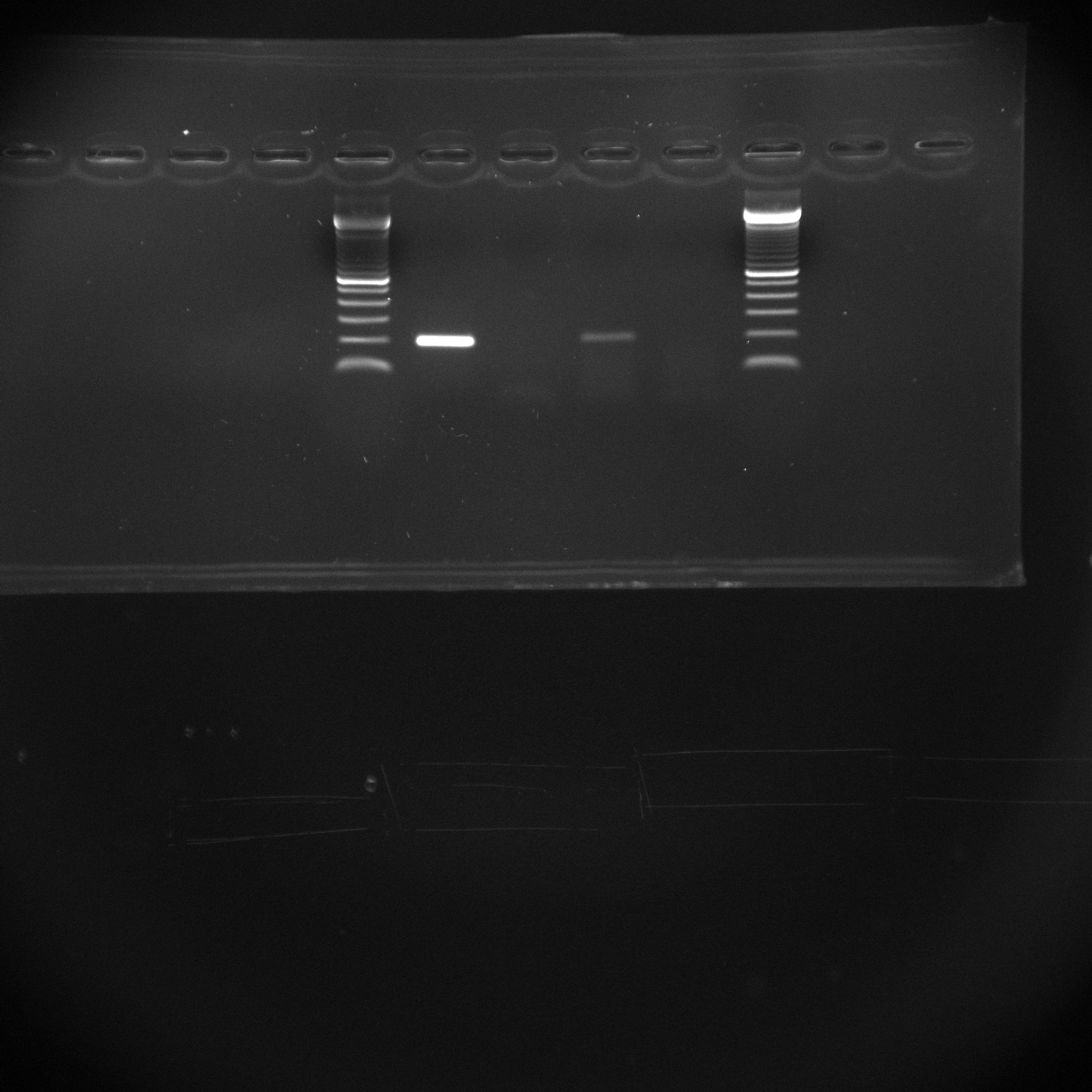

Supplement: Supplementary file 4 — Supplementary Information 2. [file 41598_2024_54227_MOESM4_ESM.tif]
