# Supplementary material for: Bone marrow stromal cell-derived hepcidin has antimicrobial and immunomodulatory activities
Source: Sci Rep. 2024 Feb 17;14:3986. doi: 10.1038/s41598-024-54227-1 (PMC10874407; doi:10.1038/s41598-024-54227-1)
Supplement: Supplementary file 5 — Supplementary Table 1. [file 41598_2024_54227_MOESM5_ESM.docx]

**Supplementary Table.** The names, sources and final concentrations of antibodies and reagents used in this study.

| ***Antibodies and reagents*** | ***Catalogue #*** | ***Source*** | ***Dilution*** |
| --- | --- | --- | --- |
| CD45 | 553076 | BD Biosciences | 0.33 µg /10^6^ cells |
| CD45- APC | 17-0451-82 | eBioscience | 0.4 µg /10^6^ cells |
| CD11b | 553308 | BD Biosciences | 0.33 µg /10^6^ cells |
| CD11b - PerCPCy5.5 | 45-0112-82 | eBioscience | 0.4 µg /10^6^ cells |
| Gr-1- PE | 108408 | Biolegend | 0.4 µg /10^6^ cells |
| BioMag goat-anti-rat IgG | 310107 | Qiagen | 1 mL/ 10^7^ cells |
| Fetal Bovine Serum | S11150 | Atlanta Biologicals | 5% in1xPBSw/o Ca/Mg |
| Rabbit anti hepcidin | Ab30760 | Abcam | 1/500 |
| Rabbit anti-Ms hepcidin | IL7.4 | Intrinsic Biosciences | 3 mg/mL |
| Rabbit anti Hu hepcidin | J6/H25 | Intrinsic Biosciences | 0.187 mg/mL |
| Rabbit anti Hu hepcidin | 583 | Intrinsic Biosciences | 1 mg/mL |
| Rabbit anti Hu hepcidin | Ab81289 | Abcam | 1/100 |
| Anti -rabbit polymer HRP | 87-9263 | Invitrogen | undiluted |
| Opal-650 tyramide | FP1496A | Perkin-Elmer | 1/200 |
| Anti Hu Leptin R | BAF-497 | R&D systems | 1/100 |
| Anti c-kit | BAF1356 | R&D systems | 1/100 |
| Anti CD34 | ab81289 | Abcam | 1/100 |
| DAPI (5 mg/ml in DMF) | D1306 | Molecular Probes | 1/25000 |
| LPS (Lipopolysaccharides from E. coli 0111:B4) | L4391 | Sigma | 1µg/mL |
| Zymosan A from  Saccharomyces cerevisiae | Z4250 | Sigma | 1µg/mL |
